# Gallium Liquid Metal Microdroplets for Constructing Active Therapeutic Agents in Photothermal Therapy of Ulcerative Colitis

**DOI:** 10.3390/mi16121420

**Published:** 2025-12-18

**Authors:** Zesheng Li, Yuzhu Di, Lubo Jin, Bo Qu, Hongyue Zhang

**Affiliations:** 1 State Key Laboratory of Space Environment Interation with Matters, Harbin Institute of Technology, Harbin 150001, China; li-zesheng@hit.edu.cn; 2 Frontiers Science Center for Matter Behave in Space Environment, Harbin Institute of Technology, Harbin 150001, China; 3 Department of Gastroenterology and Hepatology, The Second Affiliated Hospital of Harbin Medical University, Harbin 150086, China

**Keywords:** liquid metal, active droplet, colloidal motor, NIR propulsion, photothermal effect, ulcerative colitis treatment

## Abstract

Gallium-based liquid metals have been extensively studied in the field of biomedical engineering, including applications in tumor and inflammatory disease therapy, as well as targeted drug delivery. Among these, leveraging the photothermal effect of gallium liquid metals enables effective treatment of heat-sensitive cells in tumor regions and enhances the diffusion capability of liquid metal microdroplets. However, research on the active treatment of ulcerative colitis (UC) using photothermal therapy with liquid metals remains unexplored. This study focuses on constructing an active composite colloidal motor based on gallium indium liquid metal alloy, using liquid metal microdroplets as the core. Through layer-by-layer assembly of polyelectrolytes, a liquid metal active droplet loaded with the drug mesalazine (5-aminosalicylic acid), named as LMAD-A was developed. Under asymmetric light fields generated by NIR-II light source irradiation, LMAD-A exhibits autonomous locomotion, achieving an effective diffusion coefficient more than 800 times greater than that of Brownian motion in liquid metal microdroplets of similar size. Furthermore, LMAD-A demonstrates phototactic behavior, moving toward the NIR light source autonomously. Through in vitro and in vivo experiments in mice, it was verified that LMAD-A can aggregate, deform, and fuse in the mouse colon under photothermal effects, leading to enhanced release of the loaded drug. In simulated treatments, LMAD-A significantly alleviated DSS-induced colitis in mice, confirming the targeted therapeutic capability of active liquid metal microdroplets as an active therapeutic agent in UC-affected regions.

## 1. Introduction

Gallium-based liquid metal materials have garnered widespread discussion as promising candidates for sustainable biomedical applications due to their liquid state at room temperature and low biological toxicity [1,2,3,4]. Gallium and its alloys can be easily fabricated into micro/nanoscale particles. Upon air exposure, a stabilizing oxide layer forms spontaneously on their surface, while the particle interior retains bulk liquid properties at ambient temperature, enabling liquid metal microdroplet (LMMD) formation [3,5]. Colloidal or micro/nanoscale LMMDs exhibit unique properties compared to macroscopic droplets. Like other colloidal motors, shape-asymmetric LMMDs under ultrasound show enhanced motion surpassing standard nanoparticle Brownian motion [6,7,8,9,10]. By introducing structural asymmetry or incorporating chemical reactions, LMMDs can serve as the foundation for constructing active colloidal motors, thereby expanding their potential for in vivo therapeutic applications such as targeted delivery [11,12,13,14,15,16,17,18,19].

Ulcerative colitis (UC), a chronic inflammatory bowel disease (IBD), poses a growing global health burden. Its incidence has risen worldwide, especially in newly industrialized Asian nations. Notably, chronic colonic inflammation significantly increases colorectal cancer (CRC) risk [20,21,22]. Controlling colonic inflammation is crucial for improving patient outcomes. Mesalazine, a first-line treatment for mild-to-moderate UC, uses pH-dependent coating for colon-targeted release. However, individual GI pH variations and inflammatory changes often cause premature coating disintegration, leading to drug release before reaching colon lesions. This results in inadequate local drug concentration, suboptimal efficacy, and frequent escalation to immunosuppressants or biologics. These alternatives are costly, risk serious adverse effects (including infections and malignancies), and exhibit primary non-response rates up to 30% for anti-TNF-α agents, leaving limited treatment options [23,24,25,26]. Inefficient targeted delivery of current mesalazine formulations is a key reason for first-line treatment failure. New strategies independent of gastrointestinal conditions are urgently required to achieve precise colon targeting. Such developments are critical to overcoming current UC therapeutic limitations.

**Figure 1:**
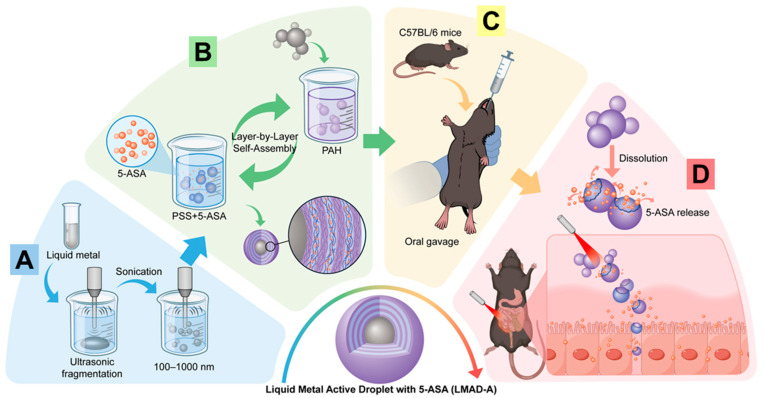
Scheme of constructing liquid metal active droplet with 5-ASA (LMAD-A) and applying LMAD-A in active therapy of ulcerative colitis. (**A**) construction of LMMD by ultrasonic fragmentation method. (**B**) LMAD-A was synthesized by LbL using PAH and PSS+5-ASA solutions. (**C**) Oral administration of LMAD-A to mice was performed by gavage means. (**D**) Under the activation of NIR laser, LMAD-A achieves active aggregation and fusion at the lesion site, promoting the drug release of the carried 5-ASA.

Herein, we present the technique route that fabricates liquid metal active droplet (LMAD) by means of layer-by-layer assembly (LbL), and the drug-loaded LMAD with specific photoactivity would be targeting deliver drug towards UC lesion, achieve the effect of targeted therapy. As a colloidal motor driven by near-infrared (NIR) laser, LMAD exhibits self-propulsion capability, and its core remains liquid as a gallium alloy, hence it is termed an active droplet. The whole presentation is shown in Figure 1. LMMD, which were synthesized via an ultrasonication crushing method, were assembled by two kinds of polyelectrolyte, poly(allylamine hydrochloride) (PAH) and poly(sodium styrene sulfonate) (PSS), this LbL process could protect LMAD from chemical environments that can easily react with liquid metal, such as succus gastricus with pH = 1, and would load 5-aminosalicylic acid (5-ASA), the main active ingredient of mesalazine into LMAD, constructing the LMAD with 5-ASA (LMAD-A). Guided by NIR-II laser irradiation, which offers superior tissue penetration and is more suitable for biological applications, LMAD-A could perform rapid phototactic locomotion towards the lesion, then fuse and deform during the gathering process in situ, rapidly release the carried small-molecule drugs at ulcerative colitis (UC) lesion sites, thereby achieving efficient and rapid suppression and treatment of UC with photothermal effect [27,28,29].

## 2. Materials and Methods

### 2.1. Materials

EGaIn alloy was purchased from Hunan Zhongcai Shengte New Materials Technology (Changde, China), PAH and PSS were purchased from Sigmaaldrich (Darmstadt, Germany), 5-ASA and anhydrous ethanol was purchased from Innochem (Beijing, China). Deionized water was produced by a Milli-Q pure water system.

### 2.2. Synthesis and Characterization of LMAD and LMAD-A

Liquid metal microdroplets (LMMD) were synthesized via sonication method, using an XM-1000T integrated ultrasonic crusher produced by Xiaomei Ultrasonic Instrument (Kunshan) Co., Ltd. (Shanghai, China). The sonication power was 900 W, with a program of 5 s working time and 5 s pause, the total sonication time was 30 min. LMAD was fabricated by layer-by-layer assembly (LbL) method with two kinds of polyelectrolyte of different charge. The two kinds of polyelectrolyte, PAH and PSS, were dissolved in 0.5 M NaCl aqueous solution, the concentration of polyelectrolyte was 2 mg/mL. About 0.05 g LMMD was first dispersed in the PSS solution, and an ultrasonic cleaner was applied to oscillate the dispersant for 10 min, and then 0.5 M NaCl aqueous solution was used to wash the LMMD-in-assembly for three times. The washing process was as follows: centrifuge the dispersant using 3500× *g* of centrifugal force, collect the precipitate and re-disperse it in the NaCl solution. After washing process, dispersed LMMD-in-assembly in the PAH solution for another 10 min. Repeat the above steps until at least three bilayers were assembled on the LMMD surface, at the sequence of PSS-PAH-PSS-PAH-PSS-PAH, that is, the LMAD was constructed. To build the LMAD-A, 5-ASA was dissolved into the PSS solution, and the mixture was applied into the synthesis program. The assembled polyelectrolyte multilayers were observed via TEM. Using the edge contrast of LMAD-A as a reference standard to distinguish LMMD from the polyelectrolytes, the total thickness of the multilayers was estimated to be 15–45 nm [30].

SEM images were obtained by Merlin Compact from Zeiss (Jena, Germany); TEM images were obtained by TALOS F200X from FEI (Hillsboro, OR, USA). X-ray diffraction (XRD) patterns were collected by EMPYREAN from PANalytical(Malvern, UK), and thermogravimetric analysis (TG) was carried out by a STA 449 F5 synchronous thermal analyzer from NETZSCH (Selb, Germany). Absorption spectrum of 5-ASA was tested by Lambda 950 spectrophotometer from PerkinElmer (Shelton, CT, USA).

### 2.3. Instrument Setup for NIR Activation and Photothermal Effects of LMAD and LMAD-A

A NIR laser of 1064 nm wavelength from Changchun Leirui Optoelectronic Technology Co., Ltd. (Changchun, China). was applied as NIR source. An IX-71 inverted biological microscope from Olympus was used to observe and record movement behavior of LMAD and LMAD-A.

The quantitative analysis of the photothermal effect of LMAD-A was conducted by measuring the temperature change in an aqueous dispersion containing a specific concentration of LMAD-A. Thermal images were captured using a Hikmicro TPK20 (Shanghai, China) infrared thermal imager, and the temperatures under thermal imaging were recorded.

### 2.4. Animal Model and Experimental Protocol

All animal experiments were conducted in accordance with the National Institutes of Health Guide for the Care and Use of Laboratory Animals and were approved by the Animal Ethics Committee of The Second Affiliated Hospital of Harbin Medical University (Approval No: SYDW2025-109). Specific pathogen-free (SPF) male C57BL/6J mice (8–10 weeks old) were obtained from Animal Experiment Center of The Second Affiliated Hospital of Harbin Medical University. All mice were housed under standard SPF conditions (12-h light/dark cycle, 22 ± 1 °C, 50 ± 10% humidity) with free access to autoclaved chow and water. The mice were randomly assigned into four groups (*n* = 6 per group): (1) Healthy Control, (2) DSS Model, (3) DSS + 5-ASA, and (4) DSS + LMAD-A. Acute colitis was induced by adding 2.0% (*w*/*v*) dextran sulfate sodium (DSS; MP, MW: 36–50 kDa) to the drinking water for 10 consecutive days. The Control group received normal drinking water. From day 11 until the end of the experiment (day 18), DSS was withdrawn, and the treatment groups received daily oral gavage of 5-aminosalicylic acid (5-ASA, [100 mg/kg]) or an equivalent dose of the 5-ASA/LMAD-A formulation. The body weight was recorded daily. The Disease Activity Index (DAI) was assessed based on a combined score of weight loss, stool consistency, and fecal blood content. Colon length was measured and photographed, and the tissues were either fixed, or the epithelium was extracted for subsequent experiments.

### 2.5. Histological Analysis

Colon tissues from each experimental group were processed for histological evaluation. After fixation in 4% paraformaldehyde and paraffin embedding, tissue sections (4 μm) were prepared. For general histopathological assessment, sections were stained with hematoxylin and eosin (H&E) following standard protocols.

To specifically identify acidic mucins in goblet cells, consecutive sections were stained with Alcian Blue (pH 2.5). Briefly, deparaffinized sections were incubated in 1% Alcian Blue 8GX (Sigma-Aldrich, Darmstadt, Germany) (in 3% acetic acid, pH 2.5) for 30 min, thoroughly rinsed in distilled water, and counterstained with 0.1% Nuclear Fast Red for 10 min. All stained sections were scanned using a high-resolution slide scanner (Pannoramic MIDI, 3DHISTECH Ltd.) (Budapest, Hungary). Histopathological scoring of H&E-stained sections was assessed inflammatory cell infiltration, crypt architecture distortion, and mucosal damage on a standardized scale [31]. For Alcian Blue-stained sections, mucin-producing goblet cells were quantified by measuring the Alcian Blue-positive area per crypt from at least 10 well-oriented crypts per animal using ImageJ software (version 1.54f, USA) (Bethesda, MD, USA).

### 2.6. Immunohistochemistry (IHC)

Consecutive paraffin-embedded colon sections were subjected to immunohistochemical staining for the macrophage marker F4/80. Briefly, after deparaffinization, antigen retrieval, and blockade of endogenous peroxidase, sections were incubated overnight at 4 °C with an anti-F4/80 primary antibody (Cell Signaling Technology, Danvers, MA, USA, dilution 1:200). Detection was performed using an HRP-conjugated secondary antibody and DAB chromogen, followed by hematoxylin counterstaining. Data are expressed as the mean percentage of DAB-positive area per field using ImageJ software (version 1.54f, Bethesda, MD, USA).

### 2.7. Flow Cytometry (FCM)

Mice were sacrificed, colons were dissected under sterile conditions: surface fat was removed, colons were rinsed thoroughly with PBS, and then cut open along the longitudinal axis. Colons were placed in pre-cooled PBS containing 2% FBS, vigorously shaken, and the supernatant was discarded; thereafter, colons were cut transversely into ~0.5 cm pieces. The tissue pieces were transferred to a 50 mL centrifuge tube containing 10 mL pre-digestion solution, incubated in a 37 °C water bath with gentle shaking for 15 min, then vigorously shaken to collect the supernatant. The collected supernatant was poured into a Petri dish, large tissue pieces were removed with tweezers, the liquid was recovered, and centrifuged at 1500 rpm for 5 min at 4 °C; the supernatant was discarded, and cells were resuspended to obtain purified colonic epithelial cells. Purified colonic epithelial cells were adjusted to 1 × 10^6^ cells/mL with PBS, and 100 μL of the suspension was stained with 5 μL Annexin V-FITC and 5 μL PI (Cell Apoptosis Detection Kit, Beyotime Biotechnology, Beijing, China) by incubating in the dark at room temperature for 15 min, followed by adding 400 μL binding buffer. Flow cytometric analysis was performed within 1 h using a flow cytometer (BD FACSCanto™ II) (BD Biosciences, Franklin Lakes, NJ, USA) with excitation at 488 nm and emission at 530 nm (Annexin V-FITC) (Beyotime, Beijing, China) and 610 nm (PI), and data were analyzed via FlowJo (Version 10.9.0) to calculate apoptotic cell percentage.

## 3. Results

**Figure 2:**
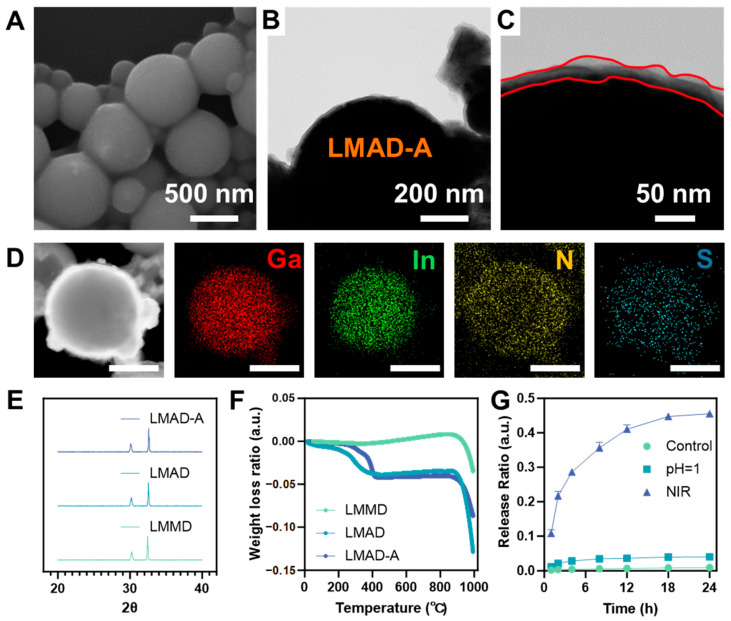
Synthesis and characterization of NIR-activated LMAD and LMAD-A. (**A**) SEM image of LMAD-A, scale bar, 500 nm. (**B**) TEM image of LMAD-A, scale bar, 200 nm. (**C**) is the enlarged image of (**B**), the irregular red lines indicate the boundaries of the polyelectrolyte multilayer, scale bar, 50 nm. (**D**) STEM image and EDS mapping images showing the distribution of Ga, In, N, and S elements in one LMAD-A, scale bar 200 nm. (**E**) XRD patterns of LMMD, LMAD and LMAD-A. (**F**) TG curves of LMMD, LMAD and LMAD-A. (**G**) Release ratio of 5-ASA loaded in LMAD-A under different conditions.

As shown in Figure 2A, the morphology of LMAD-A was observed using scanning electron microscopy (SEM). Compared to LMMD (Figure S1), LMAD-A exhibited no significant change in size, which may be attributed to the relatively compact structure of the polyelectrolyte multilayers in the dry state. The surface of LMAD-A displayed slight wrinkling compared to that of LMMD, suggesting the presence of polyelectrolyte multilayers. The diameters of LMMD and LMAD-A shown in the SEM images were statistically analyzed, with the results presented in Figure S2. The histogram indicates that the sizes of LMMD were predominantly distributed in the 500–700 nm range, accounting for 57% of the total sample population. Similarly, LMAD-A exhibited a frequency of 52% within the 500–700 nm range. Statistical analysis of droplets suitable for counting and observation in the SEM images revealed that the dry-state diameters of both LMMD and LMAD-A fell within the range of 200–800 nm, indicating that the assembly process did not induce fusion of LMMD. To further investigate the internal structure of LMAD and LMAD-A, scanning transmission electron microscopy equipped with energy-dispersive X-ray spectroscopy (STEM-EDS) was employed to characterize LMAD-A. Figure 2B,C present TEM images demonstrating the multilayer structure of LMAD-A. The images reveal an irregular edge structure with progressively varying contrast, consisting of 2–4 observable layers and a total thickness of 20–40 nm. Elemental distribution analysis of LMAD-A was performed using EDS mapping, with results shown in Figure 2D and Figure S3. Here, Figure S3 corresponds to a lower-magnification view of the area depicted in Figure 2B. In Figure 2D, signals of Ga and In confirm the presence of LMMD as the core of LMAD-A, while signals of N and S verify the successful layer-by-layer assembly of the polyelectrolytes PAH and PSS on the LMMD surface. Both Figure 2D and Figure S3 further confirm that LMAD-A possesses a shell with an average thickness of 30 nm, composed of gallium oxide and polyelectrolytes. In Figure S3, fragments of excessively oxidized LMMD were also observed. EDS mapping results indicate that regions with higher oxidation levels contain almost no In element, whereas In enrichment is detected in LMAD-A particles with only surface oxidation [32]. The signals of N and S demonstrate that the layer-by-layer assembly of polyelectrolytes remains feasible despite minor morphological variations in LMMD, which is expected to enhance the utilization efficiency of LMAD-A in the treatment of IBD.

To further investigate the phase structure of the active droplets, XRD analysis was performed on three samples—LMMD, LMAD, and LMAD-A. The obtained patterns are shown in Figure 2E. The diffraction patterns revealed characteristic peaks of Ga_2_O_3_ and In, but no distinct Ga peaks were observed. This absence may be attributed to the core of the active droplets remaining in an amorphous liquid state without a defined crystalline structure, thus preventing its detection. Thermogravimetric analysis was conducted on three samples—LMMD, LMAD, and LMAD-A—with the results presented in Figure 2F. The data indicate that, compared to LMMD, both LMAD and LMAD-A exhibited a mass loss of approximately 5% at 400 °C, indicating that the organic content in LMAD and LMAD-A accounts for about 5% of their total mass. Figure 2G presents the statistical results of the ratio of drug release amount to the total drug loading capacity of LMAD-A under three different conditions during in vitro simulated drug release, with corresponding drug release concentration data provided in Figure S4. In the acidic environment simulating gastric fluid, a drug release of 0.006 mg/mL was detected from LMAD-A after 24 h, confirming the stability of drug loading within the polyelectrolyte LbL layers. Under NIR laser irradiation, drug release from LMAD-A reached 10.8% within just 1 h, and the cumulative release ratio increased to 45.8% after 24 h, corresponding to a released drug concentration of 0.072 mg/mL in the buffer solution. Subsequent complete digestion of LMAD-A using strong acid revealed a drug loading capacity of 0.004 mg/mL of LMAD-A. The in vitro simulated drug release experiments demonstrate that LMAD-A can be administered orally, traverse the digestive tract to reach colonic inflammatory sites, and achieve locally elevated drug concentrations under NIR laser irradiation. Based on an oral administration dose of 20 μg LMAD-A per gram of body weight in mice, the anticipated drug concentration is projected to reach 0.037 μg/g.

**Figure 3:**
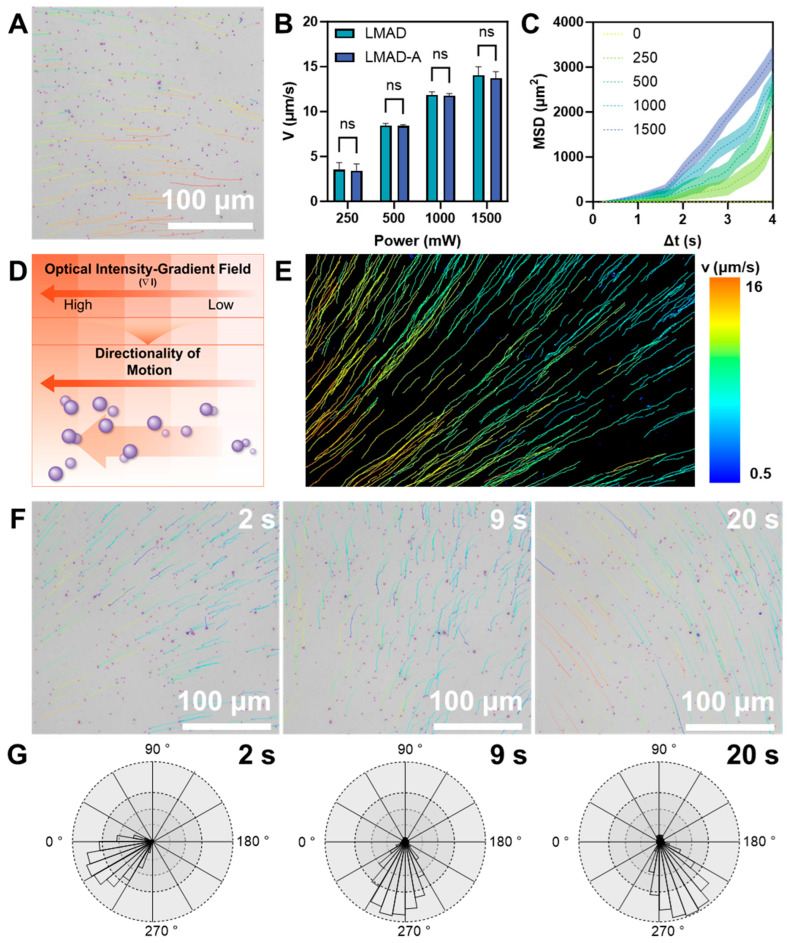
Autonomous Locomotion and phototaxis of LMAD and LMAD-A. (**A**) Microscopy image showing the straight movement of LMAD under NIR irradiation, scale bar, 100 μm. (**B**) Velocity of LMAD and LMAD-A under different NIR input power, *n* = 5, ns represents not significant (*p* > 0.05). (**C**) Mean square displacement (MSD) of LMAD under different NIR input power, *n* = 50. (**D**) Scheme of phototaxis behavior of LMAD with gradient light field. (**E**) Trajectories of LMAD activated by gradient NIR field, NIR source located at the lower left corner of vision, the pseudo-color indicates the velocity. (**F**) Time lapse images of LMAD in phototaxis behavior, the direction follows the movement of the NIR source, and the pseudo-color display shows the movement trajectory of 6 s before the snapshot, scale bar, 100 μm. (**G**) Direction distribution corresponding to the time point in (**F**).

Similarly to other active colloidal particles that exhibit characteristics induced by light stimulation, both LMAD and LMAD-A demonstrate enhanced diffusion under near-infrared light [33,34,35]. Unlike other active colloids, LMAD, due to the broad absorption properties derived from the metallic nature of liquid metal materials, can perform quasi-rectilinear autonomous motion within short time periods. As shown in Figure 3A and the corresponding Video S1, the motion records of LMAD undergoing straight locomotion under a laser input power of 1500 mW are presented, displaying the trajectory of LMAD. Under extensive NIR irradiation, the moving velocity of LMAD significantly increases. The video demonstrates that LMAD travels an average distance of 46 μm within 4 s. Furthermore, the laser input power was adjusted to systematically evaluate the velocity of LMAD and LMAD-A under varying power levels, with results summarized in Figure 3B. As the laser power increased from 250 mW to 1500 mW, the average velocity of LMAD rose from 3.54 μm/s to 14.04 μm/s, while that of LMAD-A increased from 3.42 μm/s to 13.71 μm/s. The recorded data indicated no significant difference in the NIR-powered autonomous motion between LMAD and LMAD-A. Consequently, LMAD was employed in subsequent experiments and analyses to examine the photo-induced driving behavior effect in liquid metal active colloids. Figure 3C summarizes the statistical results of the mean squared displacement (MSD) of LMAD under different laser input powers. When the input laser power was 0 W, LMAD underwent free diffusion, with its overall motion exhibiting a random walk pattern. Under this condition, the Δt-MSD curve of LMAD was linear, with a diffusion coefficient of 0.2236 μm^2^/s. As the laser input power increased, LMAD exhibited enhanced diffusion with accelerated motion characteristics, and the Δt-MSD curve adopted a quadratic function form. The effective diffusion coefficients were measured as follows: 12.59 μm^2^/s at 250 mW, 71.59 μm^2^/s at 500 mW, 141.1 μm^2^/s at 1000 mW, and 197.1 μm^2^/s at 1500 mW. The data illustrated in Figure 3C correspond to the condition where Δt is less than 4 s. For Δt greater than 4 s, as shown in Figure S5, the Δt-MSD curve returned to a linear form during the 4–6 s interval under 1500 mW laser power. Furthermore, during the 6–10 s interval, the curve remained linear but with a reduced slope, indicating that the motion of LMAD reverted to enhanced Brownian motion. Based on these experimental data, the relaxation time of LMAD is inferred to be approximately 4 s [36].

Since the diffusion rates of photoactivated active micromotors reported in the literature are generally around 50 μm^2^/s, which are lower than the autonomous locomotion of LMAD, the potential enhancement of the photoactivated active colloid framework by the material properties of gallium-based liquid metal was considered [33]. The experiments observing the driving of LMAD were conducted using an inverted biological microscope. The laser optical path was not aligned with the microscope’s optical path but was instead set up separately using an external NIR laser source. As a result, the laser beam was not perpendicular to the observation plane. Variations in the irradiation angle may be one of the reasons for the short-range directional motion of LMAD. Based on this, it is hypothesized that LMAD exhibits a phototactic characteristic, moving toward regions with stronger light intensity. To verify this, an experiment was designed to construct a continuous optical gradient with gradually varying light intensity (schemed in Figure 3D). Under the influence of this optical gradient, LMAD exhibited directional movement toward the light source, as shown in Video S2. In the video, after the light source was activated, LMAD rapidly initiated movement toward the light source, with its diffusion coefficient exceeding to above 100 μm^2^/s within 5 s. Figure 3E presents a visualized statistical analysis of the trajectories and velocity rates of LMAD within the observable field of view, with the NIR light source located in the lower-left corner. The statistical results indicate that when the average distance to the light source differed by 500 μm, the velocity of LMAD increased from around 2 μm/s to 15 μm/s, with around 7 times great increasing. Based on measurements of the experimental setup, the distance between the region of highest light intensity and the microscope’s field of view is estimated to be approximately 1 mm, indicating that the observed field of view lies within the optical gradient zone formed by the light spot. Furthermore, by altering the relative position of the light spot, we attempted to demonstrate the redirection of LMAD’s phototactic movement. As shown in Figure 3F and the corresponding Video S3, the NIR source generating the gradient light field was translated from the lower-left to the lower-right of the field of view. During this movement, LMAD maintained its phototactic motion toward the light source. As the direction of the light source changed, the trajectories of LMAD generally exhibited smooth curvature, indicating minimal disruption by thermal fluctuations. The movement directions of LMAD at different time points in Video S3 were statistically analyzed for angular distribution, with the results presented in Figure 3G. The statistical data reveals that, regardless of the relative position of the NIR source, over 90% of LMADs were oriented within a 60-degree angle directed toward the light source. Combined with the previously observed photoactivated propulsion behavior of LMAD, these results demonstrate the strong directionality of LMAD’s phototactic behavior. This directional phototaxis holds potential for attracting LMAD-A to specific NIR-irradiated sites during simulated therapeutic applications.

**Figure 4:**
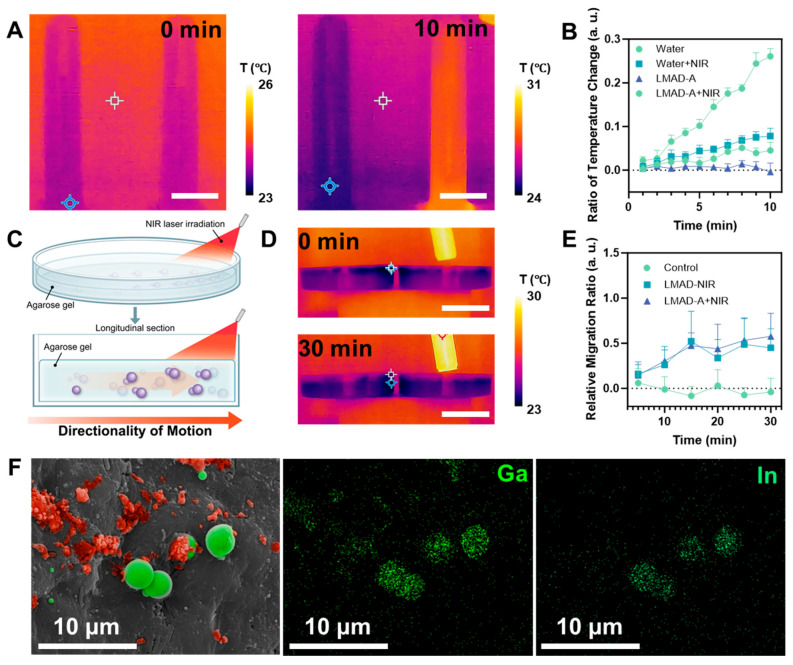
Photothermal and phototaxis applications of LMAD and LMAD-A. (**A**) Time lapse thermography images of LMAD-A dispersion irradiated by NIR, scale bar, 20 mm. (**B**) Temperature changing of LMAD-A dispersion with different NIR exposure time. (**C**) Scheme of LMAD-A migrating in agarose hydrogel with NIR activation. (**D**) Time lapse thermography images showing temperature changing of agarose gel with NIR irradiation, scale bar, 40 mm. (**E**) Relative migration ratio of LMAD and LMAD-A in agarose gel with NIR activation. (**F**) SEM image and corresponding EDS mapping of LMAD-A cluster at mice colon after LMAD-A delivery and NIR treatment, SEM image is colored by red pseudo-color indicating oxidized liquid metal particles, and green pseudo-color indicating infused, non-oxidized liquid metal particles. Scale bar, 10 μm.

After validating the photoactivated fast locomotion of LMAD and LMAD-A, further investigation was required to examine the photothermal properties and other material characteristics of LMAD-A under NIR laser irradiation to assess its suitability for in vivo therapeutic applications. Both gallium and its oxides exhibit photothermal effects under light exposure, though a clear distinction between their individual contributions has not been established due to the spontaneous formation of an oxide layer when gallium is exposed to air. Therefore, the subsequent experimental design did not differentiate the specific origin of the photothermal effect. To visually observe and measure the photothermal effect of LMAD-A, an infrared camera was used to monitor an aqueous dispersion of LMAD-A under NIR laser irradiation, as shown in Figure 4A. In the experiment, two 10 mL samples of LMAD-A dispersion at the same concentration were prepared. The right tube was irradiated with an NIR laser at an input power of 1000 mW. Over 10 min, the average temperature of the irradiated tube increased by 6 °C, while the left tube, serving as an environmental control, showed a temperature rise of only 1 °C. Additional experimental data are provided in Video S4. After adjusting the irradiation angle of the beam expander to minimize environmental interference on the control tube, the temperature difference between the two tubes increased to 4 °C after 10 min of irradiation. Building on the confirmed presence of the photothermal effect, the relative temperature changes in the LMAD-A dispersion after different durations of NIR irradiation were statistically analyzed, as summarized in Figure 4B. In the NIR-irradiated LMAD-A group, the average temperature increased by 26% after 10 min—more than four times the rate of change observed in other groups—further verifying the photothermal effect characteristic of liquid metal materials [27,37]. Considering that the proposed therapeutic procedure involves non-invasive placement of the NIR source outside the body, it was necessary to evaluate the effect of NIR penetration through tissue on LMAD-A. Prior to murine experiments, an agarose gel model was constructed, wherein LMAD-A was embedded in the lower layer and irradiated from one side with an NIR laser, as illustrated in Figure 4C. To visually demonstrate the effect of NIR irradiation on gel temperature, an experiment analogous to that in Figure 4A was conducted (Figure 4D). Two 10 cm Petri dishes containing LMAD-A and agarose gel were placed side by side, with the right dish exposed to NIR laser irradiation at 1500 mW. Results showed no significant temperature change in the agarose gel over 30 min, indicating that irradiation at this power level avoids the risk of NIR-induced burns in mice during treatment. To quantitatively analyze the effect of NIR penetration through the gel on LMAD-A, a controlled experiment was designed. After varying durations of NIR exposure, fixed regions of the gel samples were extracted, and the number of LMAD-A particles in each sample was counted. The relative change in LMAD-A quantity across groups is shown in Figure 4E. Statistical analysis revealed that, unlike the control group, which fluctuated around zero, the LMAD and LMAD-A groups exhibited change rates of 45% and 57%, respectively, after 30 min of NIR irradiation. This indicates that NIR irradiation induced migration of LMAD-A toward the illuminated region within the gel, reaching approximately 1.5 times the initial quantity. These results confirm the potential of LMAD-A to be activated by NIR light and migrate to illuminated sites even when obstructed by biological tissue. Based on these findings, an experiment was designed to locally activate LMAD-A in the mouse colon to validate its feasibility for treating colitis models. LMAD-A was administered orally to mice via gavage, and the tail base region was continuously irradiated for 30 min using an NIR laser equipped with a beam expander. Colon samples were then collected and examined by SEM, as shown in Figure 4F. EDS mapping analysis confirmed the distribution of Ga and In elements in the sample area. Furthermore, morphological distinctions revealed that partially oxidized LMAD-A (indicated by red pseudo-color in the SEM image) underwent surface oxidation and rupture, while the unoxidized core regions of LMAD-A coalesced into liquid metal microspheres with diameters of 3–5 μm (marked in green pseudo-color). Analysis of these observations indicates that under NIR irradiation, LMAD-A experienced surface oxidation and rupture, and combined with the photothermal effect of liquid metal, the contact between drug-carrying polyelectrolytes and colon tissue was enhanced. Together with the drug release data presented in Figure 2, these results demonstrate that NIR-activated LMAD-A can effectively deliver 5-ASA and enhance drug release, thereby achieving localized high drug concentrations at the disease site.

**Figure 5:**
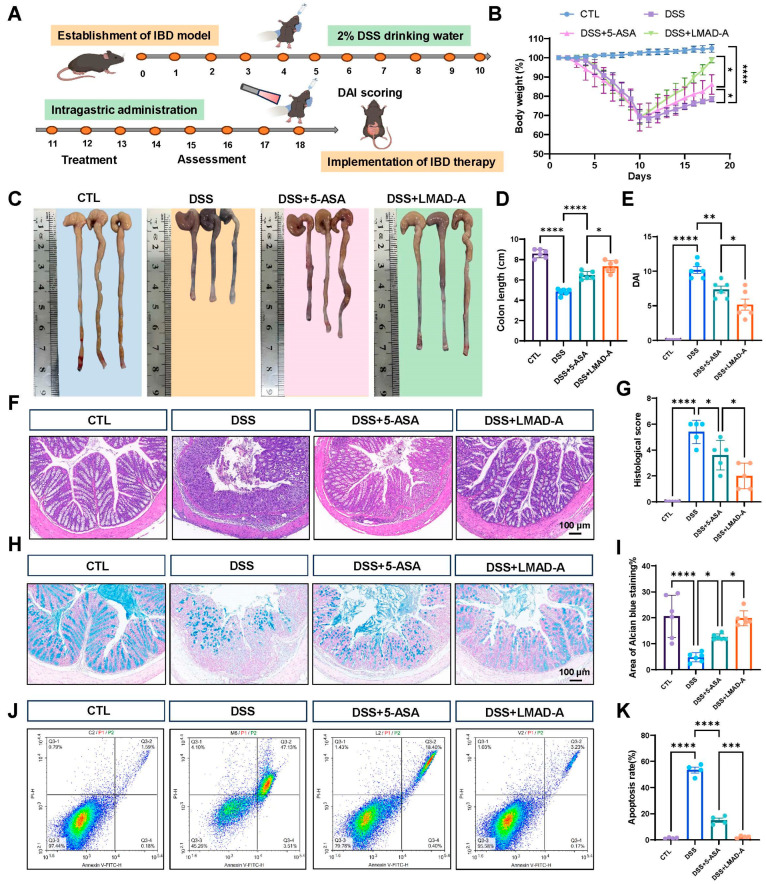
LMAD-A enhances the therapeutic efficacy of 5-ASA in a murine model of DSS-induced ulcerative colitis. (**A**) Schematic illustration of the experimental timeline for DSS-induced colitis and subsequent therapeutic interventions. (**B**) Dynamic changes in body weight ratio throughout the experimental period. Data are presented as mean ± SD (*n* = 6). * *p* < 0.05, **** *p* < 0.0001 vs. DSS or DSS+LMAD-A group. Statistical significance was determined by ordinary one-way ANOVA. (**C**) Representative macroscopic images of colons from each experimental group. (**D**) Quantitative analysis of colon length. Data are presented as mean ± SD (*n* = 6). * *p* < 0.05, **** *p* < 0.0001 vs. DSS or DSS+LMAD-A group. (**E**) Disease activity index (DAI) scores. Data are presented as mean ± SD (*n* = 6). * *p* < 0.05, ** *p* < 0.01, **** *p* < 0.0001 vs. DSS or DSS+LMAD-A group. Statistical significance was determined by ordinary one-way ANOVA. (**F**) Representative photomicrographs of H&E-stained colonic sections. Scale bar, 100 μm. (**G**) Histopathological scoring of colon tissues based on H&E staining. Data are presented as mean ± SD (*n* = 5). * *p* < 0.05, **** *p* < 0.0001 vs. DSS or DSS+LMAD-A group. (**H**) Representative Alcian blue-stained sections showing goblet cells (blue). Scale bar, 100 μm. (**I**) Quantification of the Alcian blue-positive area, reflecting goblet cell proportion. Data are presented as mean ± SD (*n* = 3, two random fields were analyzed per section). * *p* < 0.05, **** *p* < 0.0001 vs. DSS or DSS+LMAD-A group. (**J**) Flow cytometry analysis of apoptosis in colonic epithelial cells. Color denotes cell density: blue for low, green-yellow-red for increasing density (red = highest). (**K**) Quantitative analysis of apoptotic cell percentage. Data are presented as mean ± SD (*n* = 4). *** *p* < 0.001, **** *p* < 0.0001 vs. DSS or DSS+LMAD-A group.

Based on the aforementioned experimental results, a therapeutic study was designed using colitis model mice to validate the therapeutic efficacy of NIR-activated LMAD-A. To evaluate the therapeutic efficacy of LMAD-A in ulcerative colitis (UC), we established a colitis model by administering dextran sulfate sodium (DSS) in drinking water ad libitum to mice for 10 days, as illustrated in Figure 5A. This was followed by an 8-day treatment period during which the mice received either 5-ASA alone or 5-ASA loaded with LMAD via oral gavage. Body weight was measured daily throughout the experiment. As shown in Figure 5B, mice in the DSS-treated group exhibited a significant decrease in body weight ratio. Administration of 5-ASA alone resulted in a marked recovery of body weight. In contrast, treatment with LMAD-A induced a more substantial weight gain, which was statistically significant. After sacrifice, colon tissues were collected, photographed, and their lengths were measured for statistical analysis. The results (Figure 5C,D) revealed a significant reduction in colon length in the DSS group compared to the control group. This DSS-induced colon shortening was significantly reversed by 5-ASA treatment, and the therapeutic effect was further enhanced when 5-ASA was administered in combination with LMAD, leading to a more pronounced increase in colon length. Consistent findings were observed in the disease activity index (DAI) scores. DSS administration induced weight loss, increased stool frequency, led to loose stools accompanied by bleeding, and consequently elevated DAI scores. Treatment with 5-ASA reduced the DAI scores, while the combination of LMAD-5ASA further enhanced this therapeutic effect, resulting in a more significant decrease in DAI (Figure 5E).

Further histopathological analysis was performed on the middle to distal segments of the mouse colons. H&E staining (Figure 5F) showed that the DSS group exhibited loss of the colonic epithelium, destruction of crypts, and extensive infiltration of inflammatory cells. These pathological changes were markedly ameliorated by 5-ASA treatment, as evidenced by reduced inflammatory cell infiltration and partial restoration of colonic crypts. The combination therapy with LMAD-A further enhanced these improvements. A corresponding histopathological score was calculated to quantify these observations (Figure 5G). Next, Alcian blue staining results (Figure 5H,I) indicated abundant blue staining in the control group, reflecting a high proportion of goblet cells. In contrast, the DSS group showed a marked loss of blue staining. This DSS-induced reduction in goblet cells was reversed by 5-ASA treatment, and the combination with LMAD led to a further increase in the area of blue staining. Subsequently, apoptosis of colonic epithelial cells was detected. The results (Figure 5J,K) demonstrated that DSS treatment significantly increased the proportion of apoptotic epithelial cells, as shown by an increased percentage of cells in the upper right quadrant. 5-ASA treatment reduced the number of apoptotic cells, and this anti-apoptotic effect was further strengthened by the combination with LMAD-A. In summary, these results demonstrate that, compared to 5-ASA treatment alone, the combination of with LMAD and 5-ASA significantly alleviates DSS-induced colitis in mice and further enhances the therapeutic efficacy of 5-ASA.

In summary, a gallium-based liquid metal active microdroplet for drug loading (LMAD-A) was developed via LbL assembly. This autonomous active colloid is protected from gastric degradation by its LbL coating, enabling oral administration while preserving its photo-activated self-propulsion. As the LbL coating confers minimal geometric anisotropy, LMAD-A’s autonomous motion under NIR laser irradiation stems from spatially uneven irradiation. Through the photothermal effect, a temperature gradient forms on the light-facing side, generating phoretic motion. This propulsion can be amplified via structured light design, producing observable phototaxis [38,39]. The liquid metal colloidal motor fabricated via LbL assembly features a relatively simple manufacturing process, involving primarily immersion and centrifugation steps. When integrated with centrifugation and agitation equipment equipped with automated sample-handling capabilities, the production rate of LMAD-A is expected to be significantly accelerated compared to laboratory-scale conditions, thereby ensuring sufficient supply for potential clinical trials.

Photothermal-driven targeted drug release by nanocarriers at inflammatory sites offers a promising strategy for colonic diseases. Unlike other photothermal nanoparticles, LMAD-A leverages liquid metals’ broad absorption, enabling low-power actuation for deformation and fusion in biological systems. Gallium oxidation and droplet deformation synergistically enhance drug release, and gallium ions released from the liquid metal suppress bacteria and reactive oxygen species at inflammatory sites, aiding colitis treatment in mice. Colloidal motors located in the intestines or bladder remain confined to the lumen without systemic exposure [40]. LMAD-A and its fusion products would be ultimately excreted via feces, demonstrating little impact on the survival of mice.

## Data Availability

The original contributions presented in this study are included in the article/Supplementary Material. Further inquiries can be directed to the corresponding authors.

## Supplementary Materials

The following supporting information can be downloaded at: https://www.mdpi.com/article/10.3390/mi16121420/s1, Figure S1. SEM image of LMMD. Figure S2. Diameter distribution of (A) LMMD and (B) LMAD-A. Figure S3. STEM image and EDS mapping of LMAD-A. Figure S4. Concentration of 5-ASA in the drug release experiment. Figure S5. Mean square displacement of LMAD under different NIR input power, time intervals up to 10 s, *n* = 50. Video S1. The autonomous locomotion of LMAD. Video S2. The phototaxis behavior of LMAD. Video S3. The phototaxis behavior of LMAD with NIR laser source displacement. Video S4. Infrared imaging results showing temperature changes in the dispersed system containing LMAD-A. The video is played at 10× speed.

## References

[1] Wang D.W., Xu W., Liu Y.X., Chen L.Y., He T., Tan H., He S.T., Zhu J., Wang C.T., Yu Z.Y. (2025). Liquid Metal Gallium Pharmaceuticals. Theranostics.

[2] Handschuh-Wang S., Wang T., Gancarz T., Liu X., Wang B., He B., Dickey M.D., Wimmer G.W., Stadler F.J. (2024). The Liquid Metal Age: A Transition From Hg to Ga. Adv. Mater..

[3] Lin Y., Genzer J., Dickey M.D. (2020). Attributes, Fabrication, and Applications of Gallium-Based Liquid Metal Particles. Adv. Sci..

[4] Truong V.K., Hayles A., Bright R., Luu T.Q., Dickey M.D., Kalantar-Zadeh K., Vasilev K. (2023). Gallium Liquid Metal: Nanotoolbox for Antimicrobial Applications. ACS Nano.

[5] Li Z., Xu J., Wu Z., Guo B., He Q. (2022). Liquid Metal Swimming Nanorobots. Acc. Mater. Res..

[6] Li Z., Zhang H., Wang D., Gao C., Sun M., Wu Z., He Q. (2020). Reconfigurable Assembly of Active Liquid Metal Colloidal Cluster. Angew. Chem. Int. Ed..

[7] Gao Z.X., Yang Z.L., Luo M., Pei Z.Y., Xu W.T., Liu Y.S., Guo J., Xiang X., Yu Z.L., Zhao S.L. (2025). Trienzyme-in-One Nanoparticle Making Multifunctional Synergistic Nanorobot for Tumor Therapy. Small Methods.

[8] Gao Z.X., Yang Z.L., Xu W.T., Luo M., Guan J.G. (2025). Injectable nanorobots for precision cancer therapy: Motion-enhanced drug delivery. Chem. Soc. Rev..

[9] Hao X.M., Wu J.M., Luo M., Zhai X.X., Liu Y., Gao Z.X., Liu Y.R., Song Z.Y., Zhao S.L., Guan J.G. (2025). Mild photothermal-driven nanorobots for infected wound healing through effective photodynamic therapy and wound microenvironment remodeling. Chem. Eng. J..

[10] Zhai X.X., Liu Y., Hao X.M., Luo M., Gao Z.X., Wu J.M., Yang Z.L., Gan Y., Zhao S.L., Song Z.Y. (2025). Photothermal-Driven α-Amylase-Modified Polydopamine Pot-Like Nanomotors for Enhancing Penetration and Elimination of Drug-Resistant Biofilms. Adv. Healthc. Mater..

[11] Shen Y., Jin D., Li T., Yang X., Ma X. (2024). Magnetically Responsive Gallium-Based Liquid Metal: Preparation, Property and Application. ACS Nano.

[12] Wu X., Fang H., Ma X., Yan S. (2023). Gallium-Based Liquid Metals: Optical Properties, Applications, and Challenges. Adv. Opt. Mater..

[13] Chen C.R., Ding S.C., Wang J.S. (2024). Materials consideration for the design, fabrication and operation of microscale robots. Nat. Rev. Mater..

[14] Kim J., Mayorga-Burrezo P., Song S.J., Mayorga-Martinez C.C., Medina-Sánchez M., Pané S., Pumera M. (2024). Advanced materials for micro/nanorobotics. Chem. Soc. Rev..

[15] Kim K.E., Balaj R.V., Zarzar L.D. (2024). Chemical Programming of Solubilizing, Nonequilibrium Active Droplets. Acc. Chem. Res..

[16] Liu W., Liu Y., Li H., Nie H.M., Tian M.Y., Long W. (2023). Biomedical Micro-/Nanomotors: Design, Imaging, and Disease Treatment. Adv. Funct. Mater..

[17] Liu X.J., Jing Y.Z., Xu C.X., Wang X.X., Xie X.P., Zhu Y.H., Dai L.Z., Wang H.C., Wang L., Yu S.M. (2023). Medical Imaging Technology for Micro/Nanorobots. Nanomaterials.

[18] Shakya G., Cattaneo M., Guerriero G., Prasanna A., Fiorini S., Supponen O. (2024). Ultrasound-responsive microbubbles and nanodroplets: A pathway to targeted drug delivery. Adv. Drug Deliv. Rev..

[19] Wang Y., Shen J., Handschuh-Wang S., Qiu M., Du S.W., Wang B. (2023). Microrobots for Targeted Delivery and Therapy in Digestive System. ACS Nano.

[20] Ng S.C., Shi H.Y., Hamidi N., Underwood F.E., Tang W., Benchimol E.I., Panaccione R., Ghosh S., Wu J.C.Y., Chan F.K.L. (2017). Worldwide incidence and prevalence of inflammatory bowel disease in the 21st century: A systematic review of population-based studies. Lancet.

[21] Al-Horani R., Spanudakis E., Hamad B. (2022). The market for ulcerative colitis. Nat. Rev. Drug Discov..

[22] Villablanca E.J., Selin K., Hedin C.R.H. (2022). Mechanisms of mucosal healing: Treating inflammatory bowel disease without immunosuppression?. Nat. Rev. Gastroenterol. Hepatol..

[23] Steinsbø Ø., Aasprong O.G., Aabakken L., Karlsen L.N., Grimstad T. (2025). Fecal Calprotectin Correlates With Disease Extent but Remains a Reliable Marker of Mucosal Healing in Ulcerative Colitis. Off. J. Am. Coll. Gastroenterol.|ACG.

[24] Li P. (2025). Risankizumab for Ulcerative Colitis. JAMA.

[25] Sharma K., da Silva B.C., Hanauer S.B. (2025). The role of immunogenicity in optimizing biological therapies for inflammatory bowel disease. Expert Rev. Gastroenterol. Hepatol..

[26] Papamichael K., Casteele N.V., Ferrante M., Gils A., Cheifetz A.S. (2017). Therapeutic Drug Monitoring During Induction of Anti–Tumor Necrosis Factor Therapy in Inflammatory Bowel Disease: Defining a Therapeutic Drug Window. Inflamm. Bowel Dis..

[27] Fu M., Shen Y., Zhou H., Liu X., Chen W., Ma X. (2023). Gallium-based liquid metal micro/nanoparticles for photothermal cancer therapy. J. Mater. Sci. Technol..

[28] Huang Y., Wu C., Chen J., Tang J. (2024). Colloidal Self-Assembly: From Passive to Active Systems. Angew. Chem.-Int. Ed..

[29] Li H., Peng F., Yan X., Mao C., Ma X., Wilson D.A., He Q., Tu Y. (2023). Medical micro- and nanomotors in the body. Acta Pharm. Sin. B.

[30] Feldötö Z., Varga I., Blomberg E. (2010). Influence of Salt and Rinsing Protocol on the Structure of PAH/PSS Polyelectrolyte Multilayers. Langmuir.

[31] Erben U., Loddenkemper C., Doerfel K., Spieckermann S., Haller D., Heimesaat M.M., Zeitz M., Siegmund B., Kuehl A.A. (2014). A guide to histomorphological evaluation of intestinal inflammation in mouse models. Int. J. Clin. Exp. Pathol..

[32] Lin Y., Liu Y., Genzer J., Dickey M.D. (2017). Shape-transformable liquid metal nanoparticles in aqueous solution. Chem. Sci..

[33] Su Y., Xu G., Wu W., Li X., Chen S., Hong S., Lin X. (2025). Recent Advancements in Near-Infrared Light-Propelled Nanomotors for Biomedical Applications. ACS Biomater. Sci. Eng..

[34] He L., He T., Yang Y., Chen X.-B. (2025). Material selection, preparation, driving and applications of light-driven micro/nano motors: A review. Nanoscale.

[35] Deng Z., Mou F., Tang S., Xu L., Luo M., Guan J. (2018). Swarming and collective migration of micromotors under near infrared light. Appl. Mater. Today.

[36] Wan H., Xu D., Wang W., Cheng Y., Dai X., Jin X., Gao L., Zhang X., Miao B., He Q. (2024). Nonequilibrium Dynamic Phase Diagram for Transmembrane Transport of Active Particles. ACS Nano.

[37] Yan J.J., Zhang X.D., Liu Y., Ye Y.Q., Yu J.C., Chen Q., Wang J.Q., Zhang Y.Q., Hu Q.Y., Kang Y. (2019). Shape-controlled synthesis of liquid metal nanodroplets for photothermal therapy. Nano Res..

[38] Ma Z., Yang M., Ni R. (2020). Dynamic Assembly of Active Colloids: Theory and Simulation. Adv. Theory Simul..

[39] Yu N., Lou X., Chen K., Yang M. (2019). Phototaxis of active colloids by self-thermophoresis. Soft Matter.

[40] Xu D.D., Hu J., Pan X., Sánchez S., Yan X.H., Ma X. (2021). Enzyme-Powered Liquid Metal Nanobots Endowed with Multiple Biomedical Functions. Acs Nano.

