# Supplementary material for: Gallium Liquid Metal Microdroplets for Constructing Active Therapeutic Agents in Photothermal Therapy of Ulcerative Colitis
Source: Micromachines (Basel). 2025 Dec 18;16(12):1420. doi: 10.3390/mi16121420 (PMC12734736; doi:10.3390/mi16121420)
Supplement: Supplementary file 1 [file micromachines-16-01420-s001.zip › micromachines-3984687-SI.pdf]

Gallium Liquid Metal Microdroplets for Constructing Active Therapeutic Agents in Photothermal Therapy of Ulcerative Colitis

Video S1. The autonomous locomotion of LMAD.

Video S2. The phototaxis behavior of LMAD.

Video S3. The phototaxis behavior of LMAD with NIR laser source displacement.

Video S4. Infrared imaging results showing temperature changes in the dispersed system containing LMAD-A. The video is played at 10x speed.

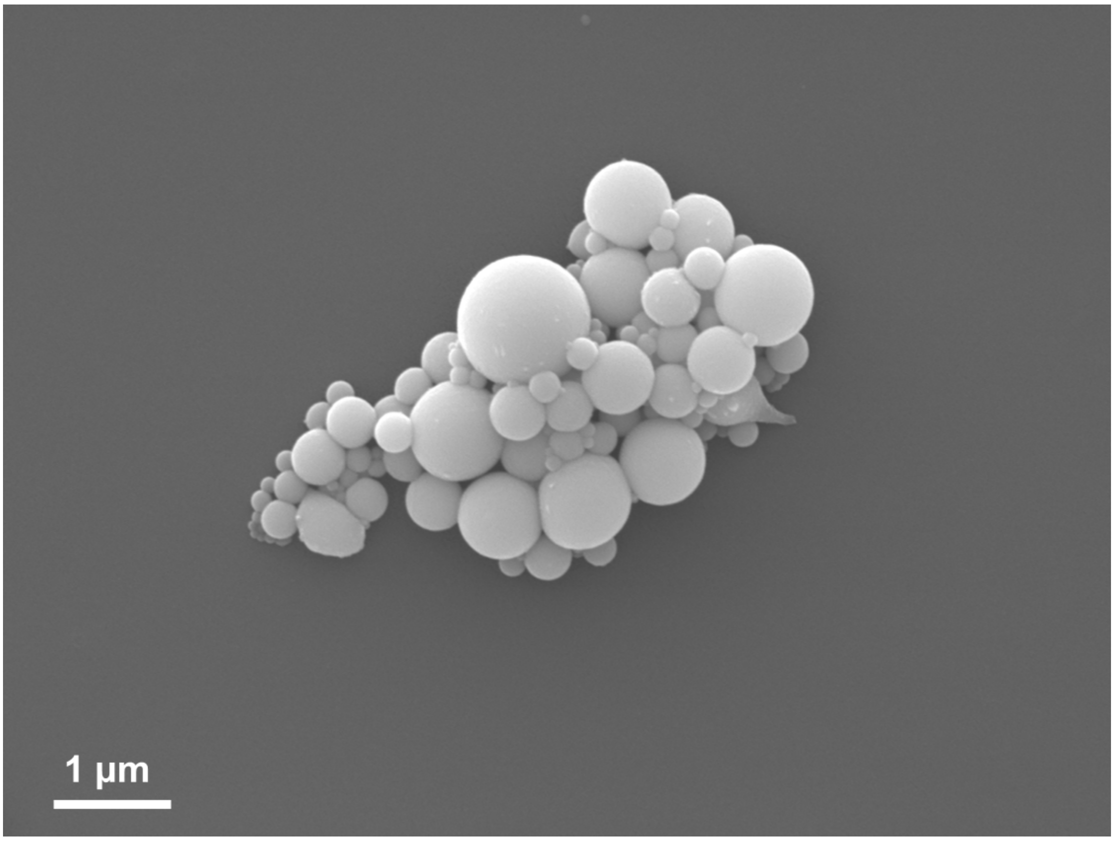

Figure S1. SEM image of LMMD.

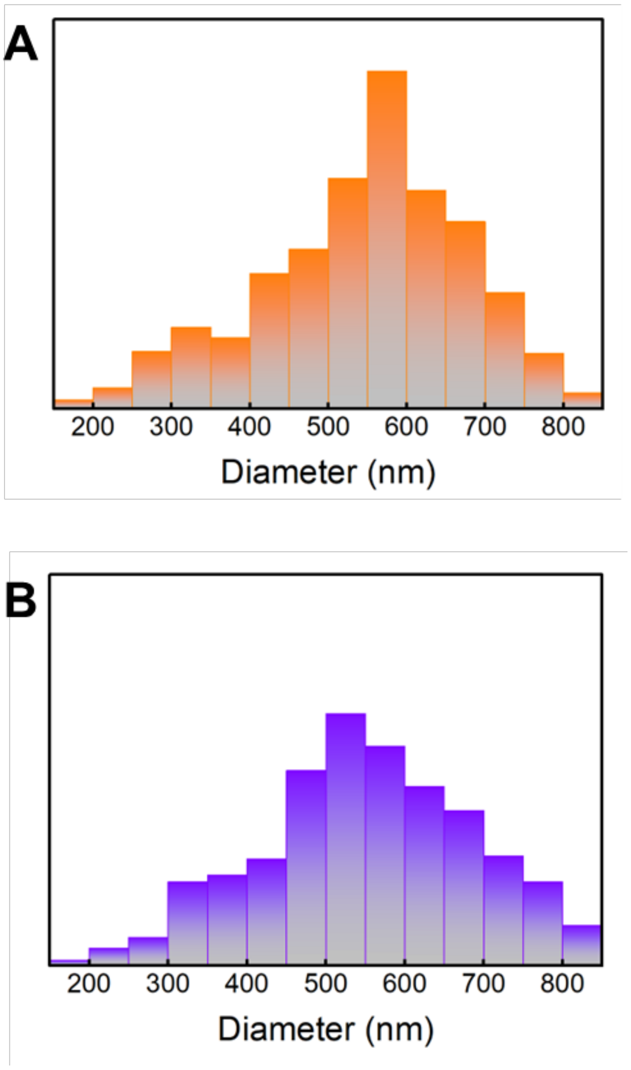

Figure S2. Diameter distribution of (A) LMMD and (B) LMAD-A.

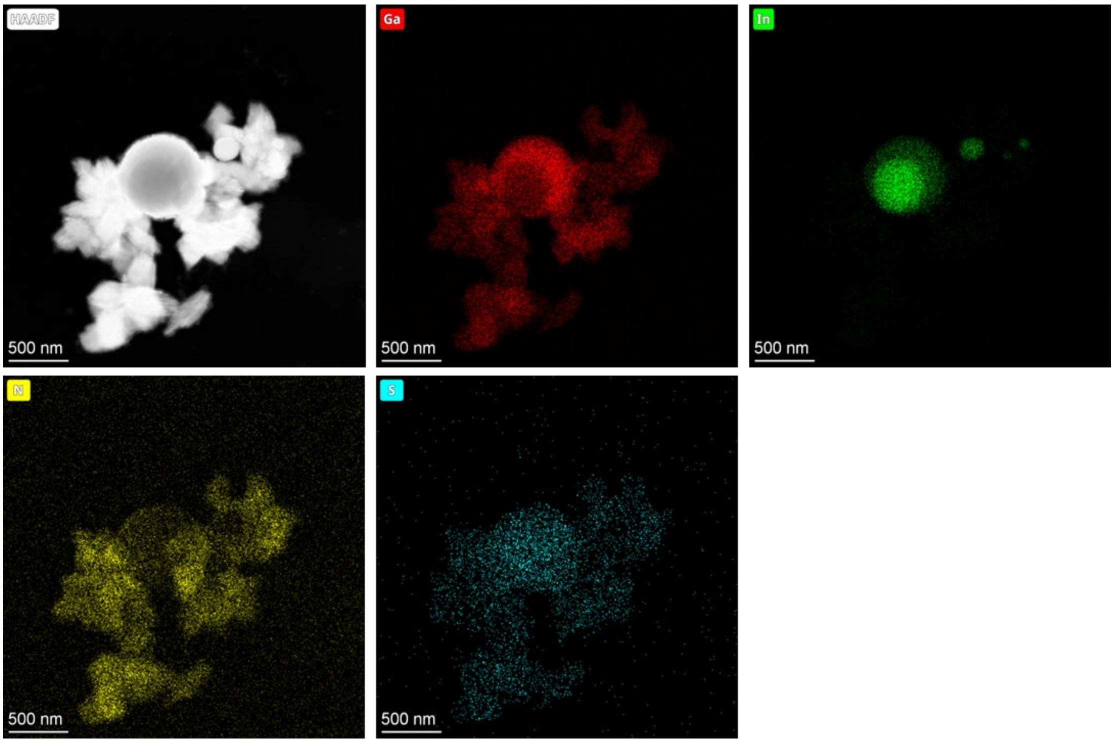

Figure S3. STEM image and EDS mapping of LMAD-A.

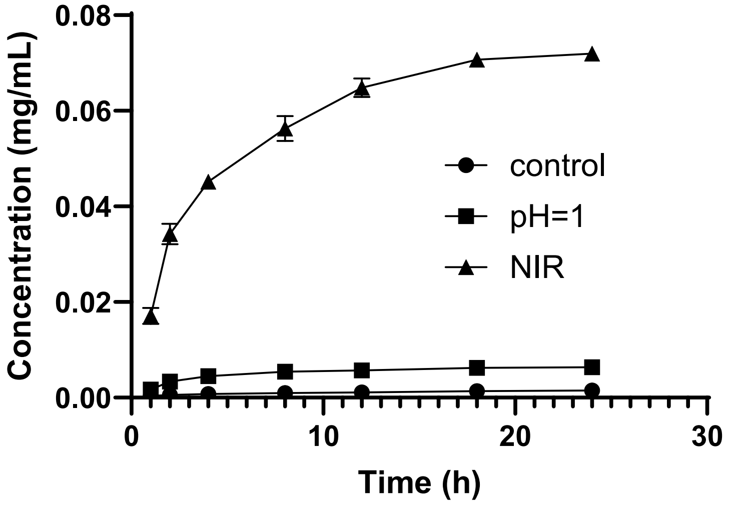

Figure S4. Concentration of 5-ASA in the drug release experiment.

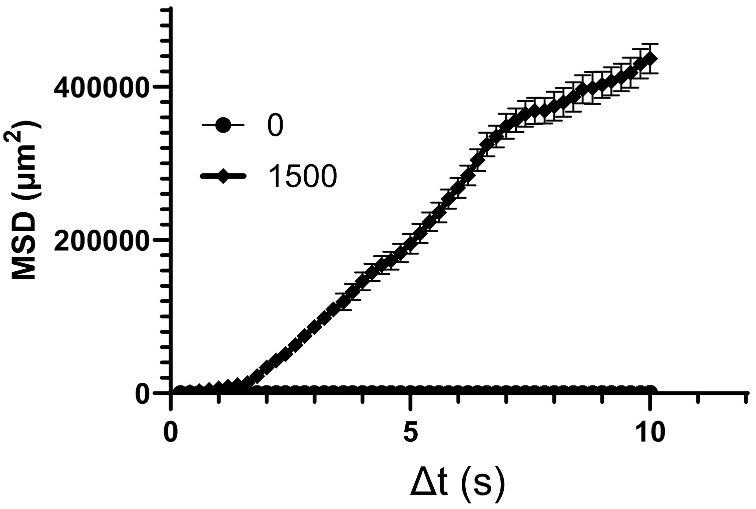

Figure S5. Mean square displacement of LMAD under different NIR input power, time intervals up to 10 s, N=50.
